# Brief screening for co-occurring disorders among women entering substance abuse treatment

**DOI:** 10.1186/1747-597X-1-26

**Published:** 2006-09-07

**Authors:** Alisa K Lincoln, Jane M Liebschutz, Miriam Chernoff, Dana Nguyen, Hortensia Amaro

**Affiliations:** 1 Department of Social and Behavioral Sciences, Boston University School of Public Health, Department of Psychiatry, Boston University School of Medicine, 715 Albany Street, T246W, Boston, MA, 02118, USA; 2 Section of General Internal Medicine (Clinical Addiction Research and Education Unit), Department of Medicine, Boston University School of Medicine, Department of Social and Behavioral Sciences, Boston University School of Public Health,, and Boston Medical Center, 91 East Concord Street, Suite 200, Boston, MA, 02118, USA; 3 Institute on Urban Health Research, Bouve College of Health Sciences, Northeastern University, 360 Huntington Avenue, Stearns Suite 503, Boston, MA, 02115, USA; 4 Potkin Research Division, Bldg,3, Rt.88, Rm 305, University of California – Irvine, Irvine, CA, 92697, USA

## Abstract

**Background:**

Despite the importance of identifying co-occurring psychiatric disorders in substance abuse treatment programs, there are few appropriate and validated instruments available to substance abuse treatment staff to conduct brief screen for these conditions. This paper describes the development, implementation and validation of a brief screening instrument for mental health diagnoses and trauma among a diverse sample of Black, Hispanic and White women in substance abuse treatment. With input from clinicians and consumers, we adapted longer existing validated instruments into a 14 question screen covering demographics, mental health symptoms and physical and sexual violence exposure. All women entering treatment (methadone, residential and out-patient) at five treatment sites were screened at intake (N = 374).

**Results:**

Eighty nine percent reported a history of interpersonal violence, and 70% reported a history of sexual assault. Eighty-eight percent reported mental health symptoms in the last 30 days. The screening questions administered to 88 female clients were validated against in-depth psychiatric diagnostic assessments by trained mental health clinicians. We estimated measures of predictive validity, including sensitivity, specificity and predictive values positive and negative. Screening items were examined multiple ways to assess utility. The screen is a useful and valid proxy for PTSD but not for other mental illness.

**Conclusion:**

Substance abuse treatment programs should incorporate violence exposure questions into clinical use as a matter of policy. More work is needed to develop brief screening tools measures for front-line treatment staff to accurately assess other mental health needs of women entering substance abuse treatment

## Background

Women present for substance abuse treatment with a diverse set of complex medical and social problems, including mental health disorders and psychological distress, high rates of history of trauma and interpersonal violence, medical problems, few vocational skills, low income, and substantial addiction severity [1-4]. General data for men and women seeking treatment for alcohol and cocaine problems show that 56–78% of clients have a lifetime psychiatric disorder and 65–73.5% have a current disorder in addition to substance use [5,6]. Estimates of the prevalence of diagnosed post-traumatic stress disorder (PTSD) range from 20 to 60% among women in substance abuse treatment [7-9]. Even more common is exposure to interpersonal violence, which we define in this paper as physical or sexual violence occurring during childhood, and/or adulthood. For example, it has been reported that more than 70% of women in substance abuse treatment report a history of childhood sexual abuse [1,4,10,11].

Among people in substance abuse treatment the co-occurrence of psychiatric distress or disorder has been associated with lower social functioning, decreased quality of life and poorer general health [12,13]. In addition people with co-occurring psychiatric disorders in substance abuse treatment have been found to have worse treatment adherence, and worse substance abuse treatment outcomes than people without co-occurring psychiatric disorders [14-16]. Among women in substance abuse treatment studies have reported that women with addiction disorders and co-occurring PTSD are more likely to have additional problems such as homelessness, criminal activity, and unemployment than peers without PTSD [5,17,18]. These social problems, along with the additional psychopathology among women in substance abuse treatment, have been associated with higher levels of impairment [5] and poor substance abuse treatment outcomes [1,19-24]. Importantly, treatments for psychiatric disorders developed for non-substance abusing patients are effective both for treating the psychiatric symptoms and for improving substance abuse treatment outcomes [25] Therefore detection of co-occurring problems among women in entering substance abuse treatment allows for the earlier referral to appropriate treatment, and thus may positively impact treatment outcomes.

This problem of co-occurring psychiatric disorders creates tremendous challenges for substance abuse treatment counselors and other front-line substance abuse treatment staff who may not be adequately trained to assess and treat trauma and mental health problems. In most substance abuse treatment programs full psychiatric assessments remain difficult to administer due to prohibitive cost and inadequate staffing despite high prevalence of co-occurring psychiatric disorders. However the evidence shows that more than half of the women with whom substance abuse counselors work are struggling with co-occurring psychiatric disorders. The lack of a practical, brief, validated screen to identify potential problems presents one barrier to improving identification of clients with needs for referral to mental health and trauma-specific programs.

A brief screen should ideally have a low false negative rate since the consequences of failing to identify a woman in need of mental health services with a substance abuse treatment setting are many. Ideally it will also produce few false positives as the resources available to substance abuse treatment facilities for further assessment and treatment are highly limited. Although there are existing instruments that assess mental health status or traumatic events, each one has different limitations for use in this setting. Kessler and colleagues developed a 6 question instrument, the K6, for inclusion in epidemiological studies to estimate the prevalence of serious mental illness (DSM-IV diagnosis in past 12 months accompanied by serious impairment) [26]. It purposely excluded impairments from substance use disorders, so its value in a substance abuse treatment setting is unclear. As well, it has not been tested in clinical settings where there is an increased prevalence of mental illness. Other instruments designed for research purposes, such as the Diagnostic Interview Schedule (DIS) [27], Structured Clinical Interview for DSM (SCID) [28] and Composite International Diagnostic Interview- Short form (CIDI-SF) [29], are either too long or require trained interviewers, making them impractical for use by front-line substance abuse treatment staff. Symptom checklists such as the Brief Symptom Inventory (BSI) are limited by their self-report of specific symptoms within the past week. In addition the BSI is expensive to administer and requires a high level of training for reliable use [30]. Zimmerman and colleagues developed the 139 question Psychiatric Diagnostic Screening Questionnaire for self-administration by clients in mental health outpatient settings. It includes 13 subscales on common psychiatric diagnoses, including common disorders such as depression, PTSD, anxiety, psychosis, and eating disorders[31]. Subsequently, the developers showed it to be valid among clients with substance use disorders[32]. The scoring algorithm requires computation of each item in the scale, and may be cumbersome for use[33]. Another measure of multiple psychiatric diagnoses, the MINI, is a 15–20 minute structured interview for multiple psychiatric disorders developed for psychiatric outpatient as well as general medical populations[34]. A short (5 minute) version, a subset of questions on depression, anxiety, eating disorders and substance abuse was developed for primary care, although was not validated in that form. The shortened MINI as well as two other similar instruments, the PRIME MD [35] and the SDDS-PC [36] have not been validated for use in substance abuse treatment settings. In fact, the presence of substance use disorders may interfere with the accuracy of some screening tests.

In addition to problems with screening generally for mental health disorders, currently available screens for posttraumatic stress disorder, an important comorbid condition for women in substance abuse treatment include 15 or more questions, are generally designed for research purposes, involve a complicated scoring algorithm, are proprietary and/or require a level of training which serves as a barrier to their use in substance abuse treatment settings [37-44].

Thus, one barrier to more comprehensive screening for co-occurring problems among women in substance abuse treatment is the lack of a valid screening tool that can be easily and quickly administered by substance abuse counselors.

In this paper we describe our efforts to develop, implement, and validate of a brief screening instrument designed to help front-line substance abuse treatment staff, primarily intake and substance abuse counselors, identify potential problems in women seeking residential and outpatient substance abuse treatment. The Boston Consortium of Services for Families in Recovery (BCSFR) Screen is designed specifically to be used by front-line substance abuse treatment staff to screen for possible PTSD and other mental health disorders. We examine whether the mental health screening items predict psychiatric diagnoses and whether asking women about their trauma histories might serve as a screen for PTSD among women in substance abuse treatment.

## Materials and methods

### Development of co-occurring disorders screen

The Boston Consortium of Services for Families in Recovery (BCSFR), a program of the Boston Public Health Commission, was the Boston site (Hortensia Amaro, principal investigator) of the SAMHSA-funded Women, Co-occurring Disorders and Violence Study [45]. The BCSFR was designed as an intervention to incorporate trauma and mental health treatment into existing outpatient and residential substance abuse treatment programs [46]. The BCSFR was a multi-site, multi-agency collaboration among providers of services, consumers, researchers and other stakeholders working with women in urban public substance abuse treatment. Soon into the implementation of the BCSFR intervention, the group identified a gap in the resources available to the front-line treatment staff: the lack of an easy to administer, brief screen to quickly identify multiple problems women faced as they entered substance abuse treatment. Thus, a committee composed of service providers, researchers, and client representatives developed screening questions for use in both substance abuse treatment and other health care settings to identify which clients might need further assessment by mental health professionals. The instrument domains included the following: demographics, mental health symptoms, interpersonal violence exposure, substance abuse, food and housing vulnerability, parenting stress, and child exposure to violence. For each area, a literature search was conducted to identify validated publicly available instruments. To minimize the length of the entire instrument, the development group examined each existing instrument for the fewest number of questions possible. In some cases, the group, with input from specialty professionals, adapted or combined existing questions.

The BCSFR Screen was developed over six months with iterative input from clients, clinicians (substance abuse, mental health, and medical), and researchers. Further revisions were made following a pilot period at each of the participating substance abuse treatment sites. During pilot testing, intake counselors at four participating substance abuse treatment programs were trained in administration of the screen and were provided materials for appropriate referral and assistance. These sites represented multiple modalities of substance abuse treatment (e.g., outpatient, methadone, and residential programs). Each site administered the screen to 5 new clients. Subjective feedback from substance abuse counselors revealed a short time (approximately 5 minutes) to administer the screen and ready acceptance by clients. Minor revisions to the instrument were made based on feedback from the pilot. The BCFSR Screen was translated into Spanish and back translated into English. Bilingual substance abuse counselors administered the Spanish version to clients who preferred to speak Spanish.

The BCSFR Screen was then administered during routine assessment by clinical staff at each site with every new client. Copies of the BCSFR screen were kept in the client's record and used to guide treatment planning and clinical care. For data collection purposes, each client's identity was masked by an identification code used by the State Department of Substance Abuse Treatment in tracking clients in publicly sponsored treatment programs. The screen results were tabulated by the Boston Public Health Commission's data management program as part of quality assurance and programmatic information.

### Content areas and sources

#### Mental health symptoms

Mental health symptom questions were created to cover 4 domains deemed important by the mental health professionals advising the project: depressed mood, anxiety, psychosis, and suicidality. The question on depressed mood was adapted from 2 separate questions on the original PRIME-MD [35]. The other questions were developed from expert input by mental health professionals and adjusted based on client-representative and service-provider input.

#### Trauma exposure

Trauma questions included queries on lifetime intimate partner violence, sexual assault, and childhood exposure to violence. The 3 questions on lifetime intimate partner violence were taken directly from STaT, a 3-question screen [47]. This 3 question screening tool for lifetime intimate partner violence has been validated in emergency department and outpatient medical settings, but not in substance misusing or treatment populations.

Questions on childhood physical and sexual abuse were adapted from the Washington State Behavioral Risk Factor Surveillance Survey [48]. The wording of these 2 questions remained the same except for a decrease in age from 18 to 13 years. The question on lifetime sexual abuse was taken from a Veterans Administration Trauma questionnaire.

#### Poverty

A total of 2 questions were selected from a 10-question instrument assessing poverty [49], which focused on difficulty obtaining food and housing in the prior 2 years.

#### Parental stress and children at risk

The 3 questions designed to assess parenting stress and children's witnessing of violence were constructed with input from clinicians with expertise in pediatrics and childhood trauma. Clinicians and client representatives made substantial changes to the wording of these questions during the review process to minimize potential offensiveness to clients.

### Validation of the BCSFR screen

Three bilingual clinicians (2 at the Doctoral level and 1 at the Masters level) blinded to the BCSFR Screen results performed a semi-structured clinical diagnostic interview for mental health diagnoses including PTSD on 88 participants participating in the overall study intervention, all of whom were clients at participating substance abuse treatment programs. Participants were assessed in either English or Spanish, depending on preferred language. The resulting diagnoses were compared with the screen results for the sub-sample of 88 women. The Institutional Review Boards at Boston University Medical Center, New England Research Institutes (NERI), and Northeastern University approved the BCSFR study.

### Analysis

Descriptive analyses were performed on data for the first 374 clients screened during implementation of the BCSFR Screen. To validate the BCSFR Screen against the criterion standard for the diagnostic interview, we conducted additional analyses on the subsample of 88 women who received diagnostic assessments. Pearson Chi-square and 2-sided Student's T-tests were used to compare the characteristics of the women who were only screened and the validation samples. We first examined the inter-item reliability among the screening questions in the subsample. Cronbach's alpha was used to measure intercorrelations among the mental health items and the PTSD items. We next examined the relationship between screening items and diagnostic interview results. We examined the predictive value of the mental health symptoms screening items for predicting mental health diagnoses and the predictive value of the trauma history items for a diagnosis of PTSD. Ninety-five percent confidence intervals were constructed for sensitivity, specificity, positive and negative predictive values, and false positive and negative estimates [50]. Fisher's Exact tests were used to assess non-random association between the test and diagnostic results. Receiver operating characteristic (ROC) curve analysis was used to compute the c-statistic, the area under the ROC curve, which measures test performance. Further analyses of the predictive value of mental health symptoms screening questions were conducted among groups stratified by age at exposure to violence (childhood and adult, adult only, child only). All statistics were computed using SAS v8.2 and SPSS v13.

## Results

### Participant characteristics

Of the 374 participants screened, the mean age was 36 years (sd = 8; range 18–67), with 31% Hispanic, 29% White, 34% Black, and 6% other. Thirty-nine percent reported at least 1 time during the past year when they were not able to obtain needed food, and 53% reported difficulty affording a place to live in the past 2 years. Eighty-eight percent of clients were mothers, of whom 33% had children with physical or behavioral problems for whom they found it difficult to care. A sub-sample of 88 women were administered both the screen and diagnostic assessment were similar to the remaining woman in our study sample. However, the women who were screened (n = 286) differed statistically from those in the validation sample only in race/ethnicity,, type of treatment, and, proportion who had children. In particular, the screening sample had a higher proportion of white women (33% vs 16%) and a lower proportion of Hispanic women (26% vs 48%). They were also more likely to be in a methadone treatment center (57% vs. 11%) and less likely to be in residential treatment (17% vs. 65%) (Table 1).

**Table 1:** Demographic Characteristics of Screening and Validation Samples.

	Screening Sample (N = 286)	Validation Sample (N = 88)	P-value
Age (mean, sd)	36.3 (8.1)	34.8 (7.7)	0.114
Race (n, %)			<0.001
Hispanic	73 (26)	42(48)	
White, non Hispanic	94 (33)	14 (16)	
Black/African American, nonHispanic	99 (35)	29 (33)	
Other/Multi Race, non Hispanic	20 (7)	3 (3)	

Substance Abuse Treatment Modality			<0.001
Methadone	164 (57)	9 (11)	
Residential	48 (17)	55 (65)	
Outpatient drug-free	74 (26)	21 (25)	

Couldn't afford rent in past 2 years	146 (53)	52(60)	0.243
Couldn't obtain food in last year	105 (38)	40 (45)	0.199
Have children	247 (88)	81 (95)	0.043

Note: Validation sample size for Treatment modality was n = 85, for rent, n = 86, for children, n = 85. P-values for age, 2-sided Student's t-test and for remaining characteristics, Pearson Chi Square test. Statistical tests and degrees of freedom (df) are: Age, t-test = 1.58, df = 372; Race/ethnicity, Chi Square = 18.95 df = 3; Substance abuse modality, Chi Square 84.96, 2 df; Rent; Food; Children, Chi Square respectively = 1.36; 1.65; 4.08; df = 1.

The results of screening are presented in Table 2. Eighty-eight percent in the total sample reported at least 1 mental health symptom in the past 30 days, with rates highest for feeling depressed or anxious. A full 89% of women reported a history of intimate partner violence, 70% reported a history of sexual assault, and 61% reported that their history of abuse began when they were under the age of 13 years. Statistical comparisons suggest that the women in the screening and validation samples differed only on the item concerning threat of violence by a partner, with more women in the validation sample reporting this experience (86 vs. 75%).

**Table 2:** Screening Results of Women in Substance Abuse Treatment (N = 390).

	Screening Sample N = 286 **(n, %)**	Validation sample N = 88 **(n, %)**	P-Value
In the past 30 days have you felt sad, blue, DEPRESSED or lost interest in things you usually cared about or enjoyed?	229 (81)	74(85)	.413
In the past 30 days have you been bothered by ANXIOUS thoughts and feelings to the point that it made it hard to do your regular activities?	187(67)	64(74)	.287
In the past 30 days have you had thoughts about HURTING YOURSELF or killing yourself or others?	46 (17)	16 (18)	.709
In the past 30 days have you been BOTHERED BY THOUGHTS that interrupted you and that you had difficulty controlling?	141 (51)	50 (57)	.285

**Any Mental Health Symptoms**	**249 (89)**	**81 (92)**	**.402**

Have you ever been in a relationship where your partner has PUSHED or slapped you?	236 (83)	74 (84)	.827
Have you ever been in a relationship where your partner has THREATENED you with violence?	213 (75)	76 (86)	.025
Have you ever been in a relationship where your partner has thrown, BROKEN and punched things?	229 (81)	76 (86)	.293

**Any Intimate Partner Violence**	**251 (88)**	**82 (93)**	**.199**

Has anyone ever used FORCE or the threat of force to have sex with you against your will?	180 (65)	62 (71)	.262

**Any Sexual Assault**	**197 (70)**	**65 (75)**	**.383**

Before you were 13, was there any time when you were PUNCHED, kicked, choked, or received a more serious physical punishment from a parent or other adult?	125 (45)	45 (52)	.221
Before you were 13 did anyone ever TOUCH you in a sexual way or make you touch them in a way you did not want them to?	140 (50)	49 (58)	.196

**Any Childhood Abuse**	**170 (60)**	**60 (70)**	**.097**

**INDEPENDENT DIAGNOSTIC ASSESSMENTS (n, %)**			
Depressive Disorder		56 (64)	
Bipolar Disorder		23 (26)	
Anxiety Disorder		19 (22)	
PTSD		58 (66)	

Note: Any sexual assault summary combines items regarding force and touching. P-values are for Pearson Chi-square test of significance with 1 degree of freedom; In validation sample, one observation was missing for mental health symptom items, one to three were missing for abuse and sexual assault items. In screening sample, 4–10 observations were missing for mental health items and 2–8 for abuse and sexual assault items.

### Validation of the mental health symptoms screening items

Using Fisher's Exact test, the relationship between each mental health symptom screening item and the appropriate diagnosis was tested. None of the mental health symptoms screening items were significant predictors of specific diagnoses, either alone or in combination with other mental health screening items. Next, a test of association was conducted for each mental health symptom screening item as well as for various combinations of screening items, each with "any psychiatric diagnoses;" no significant associations were found. Finally, the mental health symptom screening items were analyzed as counts (1–4 symptoms reported). This did not increase the power of the screening questions to identify women at risk for co-occurring psychiatric disorder(s).

### Validation of the trauma history screening items for PTSD

The Cronbach's alpha coefficient for the 6 trauma history items was 0.77. Similar analyses to those described above were conducted for the trauma history questions and PTSD diagnosis (women with PTSD diagnosis n = 58; women without PTSD diagnosis n = 30). Here, significant relationships between the trauma history screening questions and PTSD diagnosis were found using the count method for the number of trauma history items endorsed. The predictive value of PTSD diagnosis increased with the number of trauma questions endorsed. For example, women who reported 4 or more trauma screen items were likely to have a PTSD diagnosis (sensitivity 78%, specificity 53%). The false positive rate was 24%. The predictive values of PTSD diagnosis for women who endorsed 4, 5, or all 6 screening items are presented in Table 3. The area under the ROC curves for these tests are modest, all under 0.70.

**Table 3:** Predictive Value of PTSD Diagnosis by the Number of Trauma Screening Items Endorsed (n = 88).

		Dx Pos.	Dx Neg.	Sensitivity (95% CI)	Specificity (95% CI)	Pred. Value Pos. (95% CI)	Pred. Value Neg. (95% CI)	C-Statistic^1^	Fisher's Exact P-value
4 PTSD Items Endorsed	Pos.	45	14	78% (67% – 88%)	53% (35% – 71%)	76% (65% – 87%)	55% (37% – 73%)	0.66	.0046
	Neg.	13	16						
5 PTSD Items Endorsed	Pos.	39	10	67% (55% – 79%)	67% (50% – 84%)	80% (68% – 91%)	51% (36% – 67%)	0.67	.0033
	Neg.	19	20						
6 PTSD Items Endorsed	Pos.	28	3	48% (35% – 61%)	90% (79% – 100%)	90% (80% – 100%)	47% (34% – 60%)	0.69	<.001
	Neg.	30	27						

Note: ^1^The c-statistic is the area under the Receiver Operating Characteristic (ROC) curve, which measures test performance.

### Interpersonal violence history and mental health symptoms in combination

At least 1 of the interpersonal violence history items was endorsed on ninety-seven percent (85 of 88) of the screens. Fifty-seven women (67%) reported experiencing both childhood and adult interpersonal violence. Twenty-four women (28%) experienced trauma only during adulthood. Similar analyses to those described above for validating the mental health screening items were conducted within these strata. In the both groups, women reporting both, childhood and adult interpersonal violence histories, the results paralleled those found among the full validation sample.

## Discussion

As expected, this sample of women entering substance abuse treatment programs reported high rates of co-occurring mental health symptoms, interpersonal violence exposure, PTSD, and other life stressors. The validation of the BCSFR Screen, with brief screening items on mental health symptoms and interpersonal violence exposure, demonstrated a strong ability to predict PTSD but no other major psychiatric disorders. The six interpersonal violence history questions provide substance abuse counselors with a strongly predictive marker for PTSD among the women they work with in a simple and easy to administer battery of questions. The questions also give the substance abuse counselor relevant clinical history.

Mental health symptoms assessed on the BCSFR Screen did not add predictive power to determine which women entering substance abuse treatment have a co-occurring psychiatric diagnosis as confirmed by an independent clinical assessment. This finding is consistent with published literature on the lack of sensitivity of a 2-question dichotomous screener for depression from the PRIME-MD [51]. Other researchers have found good test characteristics to 2 similar questions on depressive symptoms that were modified by using 4 answer choices ("not at all," "several days," "more than half the days," and "nearly every day," scored as 0, 1, 2, and 3, respectively) [52]. This could be considered in future studies of mental health symptom screening. The K6 questions [26] to discriminate serious mental illness should also be tested in substance abuse treatment settings.

Despite lack of predictive value for depression, bipolar disorder, and anxiety disorder, use of these types of screening questions may add important value to the substance abuse intake process for a number of reasons. First, substance abuse counselors in this study became aware of these symptoms, which was important to the women seeking care. For example, 17% of the women in this study reported suicidal ideation in the past 30 days. Mental health symptoms are also important predictors of substance abuse treatment outcomes [1,19-21,23-25]. In addition, asking these types of questions combined with training raises awareness among members of the substance abuse treatment team about the need to incorporate mental health and trauma factors into treatment planning. Strengthening the sensitivity through more answer choices (see above) might improve the discriminatory function while maintaining a simple and user-friendly screening tool.

The strengths of our study include the ability to generalize findings to clinical substance abuse programs, the straightforward nature of the BCSFR screening questions, and instrument validation in a substance abuse treatment population. The input of different constituencies into the development of the BCSFR Screen is also novel, as most of the other PTSD and mental health screening instruments were developed solely by research personnel and professionals. Client representatives and practicing substance abuse counselors were instrumental in the development of our entire screen, including instructions and layout of the interview instrument. While validation of the Spanish version of the instrument was limited by the small sample size, another strength of the current study was testing in both Spanish and English; other instruments are available only in English (Spanish version available from Dr. Hortensia Amaro). Study limitations include the criterion measure – a semi-structured interview by a single mental health professional. Use of an instrument such as the SCID would have strengthened the validation [28]. However, the direction of any theoretical bias is not known because the interviews were conducted in standard fashion by an impartial clinician without knowledge of the prior screening results. In addition, this study does not discern the possible effects of withdrawal/early recovery. Screening results at admission may reflect inflated depression and anxiety scores due to the combination of physiological and psychological withdrawal as well as fears and anxieties about the treatment process and its impact on self, children, and other relationships. Future work should explore the implications of these influences on mental health status for appropriate clinical care.

## Conclusion

Given the high prevalence of co-morbid mental health disorders and distress among adults in publicly funded substance abuse treatment, front-line treatment staff must have resources to quickly assess the level of need of their clients. Few such resources exist and more work is needed to develop a tool available to front-line treatment staff to help them identify which clients should be referred for further mental health evaluation. We have developed and tested a brief screen for mental health symptoms, exposure to interpersonal violence, and other social stressors. The tool, brief and easy-to-use, demonstrates good test characteristics for predicting active PTSD but is unable to predict other diagnoses. Mental health symptom questions might be modified in the future in order to improve predictive value for these other mental health diagnoses. This work is a first step toward improving the ability of front-line staff in substance abuse treatment to identify clients who might benefit from diagnostic assessment and referral for mental health services. Future work should focus on filling this gap in resources available to substance abuse treatment staff.

## Competing interests

The author(s) declare they have no competing interests.

## Authors' contributions

AL assisted participated in the development of the BCSFR screen, worked with treatment site staff to implement screening, led the efforts to conduct the validation study and wrote the first draft of the manuscript. JL led the selection and development of screen items and assisted with the development of the manuscript. MC conducted statistical analyses and assisted with the development of the manuscript. DN conducted statistical analyses. HA is the PI of the project and oversaw all aspects of the screen development and implementation, design of the study and data analyses and assisted with the development of the manuscript. All authors read and approved the final manuscript.
